# Color Changes in Gels Composed of Hydrogen‐Bonding Donor–Acceptor–Donor‐Type Fluorenone Derivatives and Short‐Chain Polyethylene Glycol in Response to Ionic Species

**DOI:** 10.1002/asia.202500129

**Published:** 2025-04-14

**Authors:** Syota Yamada, Mao Suzuki, Ken'ichi Aoki, Atsushi Seki

**Affiliations:** ^1^ Department of Chemistry Graduate School of Science Tokyo University of Science 1‐3 Kagurazaka, Shinjuku‐ku Tokyo 162‐8601 Japan; ^2^ Department of Chemistry Faculty of Science Tokyo University of Science 1‐3 Kagurazaka, Shinjuku‐ku Tokyo 162‐8601 Japan

**Keywords:** Donor–acceptor molecules, Intramolecular charge transfer, Stimuli‐responsive materials, Supramolecular gels

## Abstract

A series of donor–acceptor–donor (D–A–D)‐type fluorenone derivatives with urethane units and oligo(ethylene glycol) (OEG) linkers of different lengths (**F‐EG*_n_*‐BU**, *n* = 2–4) are synthesized to investigate the effect of the length of the OEG linker on the self‐assembling behavior and photophysical properties of the fluorenone derivatives. In alcoholic media such as ethanol and 2‐propanol, the fluorenone‐based D–A–D triad with triethylene glycol linkers (**F‐EG_3_‐BU**) specifically form supramolecular gels via hydrogen bonds between the urethane units. The extension of the OEG linkers (**F‐EG_4_‐BU**) reduced the strength of molecular aggregates, resulting in higher solubility in various organic solvents. Each compound formed intramolecular charge transfer (ICT) states, as evidenced by the ICT band in the UV–vis absorption spectra and the twisted intramolecular charge transfer emission bands in the photoluminescence spectra. Changes in the absorption and emission spectra of short‐chain poly(ethylene glycol)s (SC‐PEGs) gelated with **F‐EG_2_‐BU** depend on the coexisting ionic species. In this study, we describe the unique chemical stimuli–response behaviors of fluorenone‐based SC‐PEG gels that cause significant changes in the spectroscopic properties upon ion doping with NaCl and alkali metal hydroxides.

## Graphical abstract

Fluorenone‐based donor–acceptor–donor‐type supramolecular gelators with urethane groups separated by oligo(ethylene glycol) linkers were developed. The addition of alkali metal cations induces a significant fluorescence color change by altering the balance between the emission from the locally excited and the twisted intramolecular charge transfer states. These supramolecular gels respond to chemical stimuli and thus have strong potential as colorimetric materials.

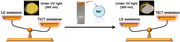

## 1 Introduction

Low‐molecular‐weight gelators (LMWGs) are representative supramolecular materials with applications in various fields, such as food industry, biomedical science, and device science, owing to their characteristic viscoelasticity, mass transport, and stimuli‐responsive properties.^[^ ^1^ ^]^ Various “π‐gelators”, LMWGs containing a conjugated π‐system. have been developed in the past few decades.^[^ ^1^, ^2^ ^]^ Supramolecular gelation in various π‐gelator systems is primarily driven by π–π interactions and physical cross‐linking resulting from intermolecular hydrogen bonds.^[^ ^1^, ^2^ ^]^ The synergistic effects of these intermolecular interactions determine the formation or decomposition of supramolecular gels in appropriate solvents.^[^ ^3^ ^]^ Accordingly, molecular‐scale variations in supramolecular gels can be translated into bulk properties,^[^ ^1^, ^4^ ^]^ enabling the development of stimuli‐responsive LMWGs.^[^ ^5^ ^]^ Various supramolecular gels that respond to chemical stimuli have been reported.^[^ ^6^ ^]^ Nanoscopic structural changes in typical ion‐responsive gel systems driven by metal coordination, intercalation, or metal templating are amplified to macroscopic phase transitions and spectral changes.^[^ ^6^, ^7^ ^]^

The introduction of a highly affinitive group to a specific chemical species provides a useful approach for developing functional LMWGs that respond to desired target species. Conventional molecular design of chemical stimuli‐responsive LMWGs involves the introduction of a functional unit into the LMWG skeleton that interacts strongly with the target species to form supramolecular assemblies.^[^ ^4b^ ^]^

Organic π‐conjugated dyes incorporating a donor–acceptor (D–A) skeleton exhibit unique spectroscopic properties owing to intramolecular charge transfer (ICT) from electron‐rich to electron‐deficient units.^[^ ^3^, ^8^ ^]^ Some D–A‐type π‐conjugated compounds exhibit characteristic fluorescence via a twisted intramolecular charge transfer (TICT) state.^[^ ^3^, ^8^, ^9^ ^]^ These D–A‐type dyes may act as fluorocolorimetric probes for the detection of external stimuli based on the ICT sensitivity to a polar environment.^[^ ^3b^ ^]^ Aromatic D–A‐type compounds are known to exhibit thermofluorochromism, solvatofluorochromism, and mechanofluorochromism.^[^ ^10^ ^]^ Thermally stable fluorenone is an attractive electron acceptor with a planar π‐conjugated system. The rigid aromatic backbone of fluorenone plays a key role in its self‐assembly behavior, while the electronic states and orbital overlap between adjacent molecules result in stimuli‐responsive color changes and electron transport in condensed states.^[^ ^11^ ^]^ Among the various fluorenone‐based functional materials, colorimetric and fluorescent chemosensors have been developed based on D–A‐type fluorenone molecules.^[^ ^11^, ^12^ ^]^

**Figure 1:**
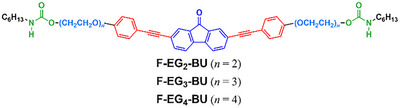
Chemical structures of hydrogen‐bonding D–A–D‐type fluorenone derivatives.

In this context, we developed stimuli‐responsive supramolecular gels of D–A‐type fluorenone derivatives.^[^ ^13^ ^]^ The aim of this study is to investigate the applicability of novel ion‐responsive π‐gelators as chemical probes. In this study, we developed ion‐responsive supramolecular gels of fluorenone‐based D–A–D triads bound to a urethane moiety via an oligo (ethylene glycol) OEG unit. Three D–A–D type fluorenone‐based bis‐urethanes with different linker lengths were synthesized (**F‐EG_2_‐BU**, **F‐EG_3_‐BU,** and **F‐EG_4_‐BU,** Figure 1) to determine the fundamental photophysical and gelation properties along with the ion‐responsive behaviors in the supramolecular gel states.

## 2 Results and Discussion

### 2.1 Photophysical Properties

**Figure 2:**
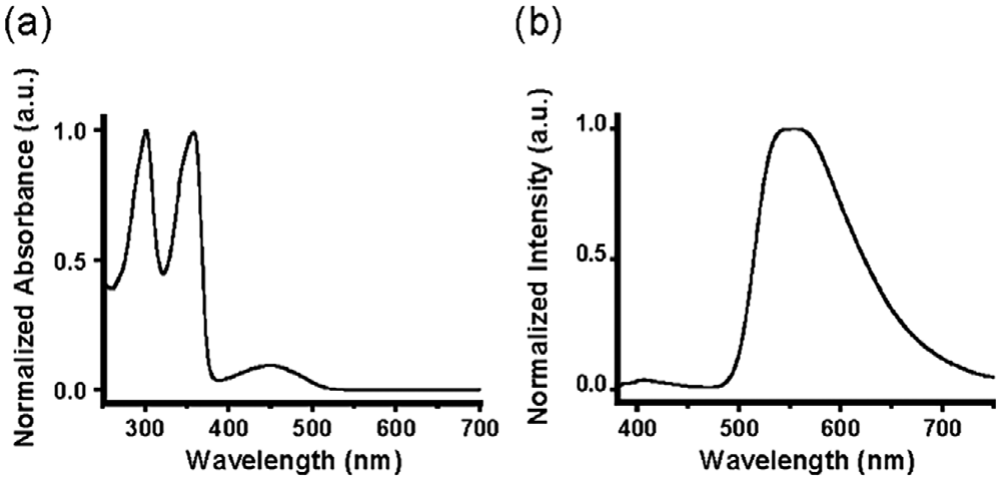
(a) Absorption and (b) emission spectra of **F‐EG_2_‐BU** in THF (10 µM).

To elucidate the electronic states of the D–A–D‐type fluorenone derivatives **F‐EG_2_‐BU**, **F‐EG_3_‐BU,** and **F‐EG_4_‐BU**, the light absorption and emission properties of dilute solutions of each derivative were measured. The absorption and emission spectra of a dilute solution of **F‐EG_2_‐BU** (10 µM) in tetrahydrofuran (THF) are shown in Figure 2a,b. The spectroscopic parameters of the THF solutions of each compound are summarized in Table S1. The absorption spectrum of **F‐EG_2_‐BU** (Figure 2a) shows two strong absorption peaks from 300 to 360 nm and a broad absorption band centered at approximately 450 nm. The former two peaks likely originate from local excitation (LE), while the latter peak is derived from the ICT transition. Each analog exhibited similarly strong emission from the TICT states regardless of the length of the OEG linker (Figure 2b). The absorption and emission peak wavelengths in the spectra of the compounds were essentially identical. The estimated photoluminescence quantum yield (PLQY) of the THF solution of **F‐EG_2_‐BU** was approximately 0.3. Compounds with longer OEG linkers (**F‐EG_3_‐BU** and **F‐EG_4_‐BU**) exhibited lower PLQYs in dilute solutions of the same concentration (Table S1).

### 2.2 Gelation Behaviors

**Figure 3:**
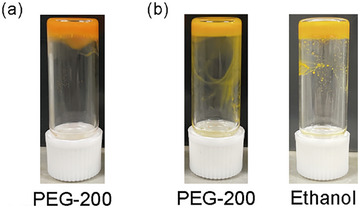
Photographs of organogels of (a) **F‐EG_2_‐BU** and (b) **F‐EG_3_‐BU** at a concentration of 50 g L^−1^.

The gelation behaviors of **F‐EG_2_‐BU**, **F‐EG_3_‐BU**, and **F‐EG_4_‐BU** were examined using a vial inversion test and are tabulated in Table 1. The fluorenone derivatives **F‐EG_2_‐BU** and **F‐EG_3_‐BU** (50 g L^−1^) formed gels in short chain poly(ethylene glycol)s (SC‐PEGs) such as PEG‐200 (average *M*_n_ ≈ 200) and PEG‐600 (average *M*_n_ ≈ 600) (Figure 3a,b). The same concentration of **F‐EG_4_‐BU** in SC‐PEGs similarly afforded a gel‐like precipitate: however, turbid gels were formed at the higher concentrations (≥ 55 g L^−1^). Notably, **F‐EG_2_‐BU** also showed gelation ability in dodecylbenzene (DB), while **F‐EG_3_‐BU** with tri(ethylene glycol) linkers also gelates lower molecular weight alcohols such as ethanol and 2‐propanol. In summary, the elongation of the OEG linkers disturbed the dense molecular packing of the fluorenone derivatives. An appropriate balance between the length of the OEG linker and strength of the intermolecular interactions may have been achieved in the **F‐EG_3_‐BU** system, thereby enabling the formation of supramolecular gels in common alcohols.

**Table 1:** Gelation properties of **F‐EG_2_‐BU**, **F‐EG_3_‐BU**, and **F‐EG_4_‐BU** at a concentration of 50 g L^−1^ (Minimum gel concentrations in g L^−1^).^a)^

Solvent	F‐EG_2_‐BU	F‐EG_3_‐BU	F‐EG_4_‐BU
*n*‐Hexane	I	I	I
Chloroform	S	S	S
Ethyl acetate	P	P	P
THF	S	S	S
Acetone	P	S	S
Acetonitrile	P	P	P
2‐Propanol	P	G (32)	WG
Ethanol	P	G (37)	WG
Methanol	P	P	P
Dodecylbenzene (DB)	G (24)	P	P
PEG‐200	G (34)	G (39)	GP
PEG‐600	G (31)	G (42)	GP
Water	I	I	I

^a)^ G, GP, I, S, P, and WG denote gel, gel‐like precipitate, insoluble, soluble, precipitate, and weak gels, respectively.

**Figure 4:**
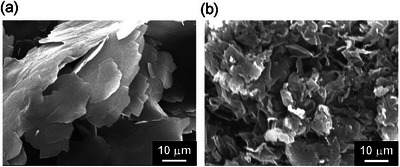
SEM images of the xerogels of (a) **F‐EG_2_‐BU** and (b) **F‐EG_3_‐BU**.

Xerogels were observed using scanning electron microscopy (SEM) to elucidate the morphology of the organogels. The flat sheet‐like objects were found in the xerogel prepared by immersing the PEG‐200 gel of **F‐EG_2_‐BU** in water (Figure 4a). In contrast, swollen objects were observed in the SEM image of PEG‐600 gel of **F‐EG_2_‐BU** due to the difficulty in removing sufficient PEG‐600 from the gel (Figure S3). In case of **F‐EG_3_‐BU**, wrinkled sheet‐like aggregates were observed in the SEM image of the xerogel sample procured from the ethanol gel (Figure 4b). Structural analysis of the SC‐PEG gels of **F‐EG_2_‐BU** and **F‐EG_3_‐BU** by X‐ray diffraction (XRD) spectroscopy was consistent with the aforementioned observations. The XRD profile of **F‐EG_2_‐BU** demonstrated the formation of layered structures in the crystalline state (Figure S4a, black line). **F‐EG_2_‐BU** in the SC‐PEG gel state exhibits a similar XRD pattern (Figure S4a, red line). Additionally, **F‐EG_3_‐BU** exhibits similar XRD patterns in the crystalline and gel states. (Figure S4b). These results implied that the morphologies of each gel were similar, forming swollen sheet‐like assemblies composed of layered structures in the gel state.

**Figure 5:**
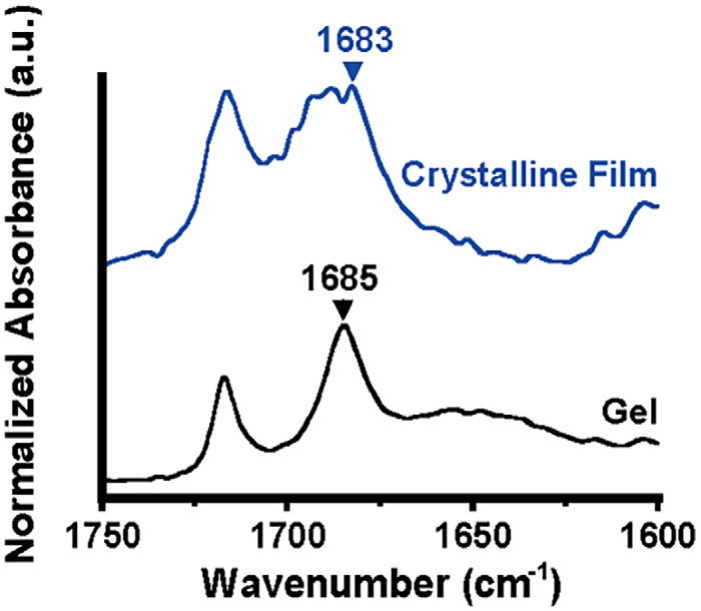
FT‐IR spectra of **F‐EG_2_‐BU** in PEG‐600 gel and crystalline film.

The bulk state of each compound was analyzed using Fourier‐transform infrared (FT‐IR) spectroscopy to clarify the contribution of intermolecular hydrogen bonds to the molecular assembly. The FT‐IR spectrum of the pristine crystalline phase of **F‐EG_2_‐BU** shows a peak at 1683 cm^−1^, which was attributed to the stretching vibration of hydrogen‐bonded C═O (Figure 5, blue line). In the FT‐IR spectrum of the PEG‐600 gel of **F‐EG_2_‐BU**, this signal was slightly shifted to 1685 cm^−1^ (Figure 5, black line), suggesting that the formation of intermolecular hydrogen bonds among the urethane moieties drives the formation of supramolecular gel networks. The FT‐IR spectrum in the DB gel of **F‐EG_2_‐BU** also supports this consideration. Although the N − H stretching vibration signal was obscured by the broad O − H stretching vibration band in the FT‐IR spectra of SC‐PEG gels, the FT‐IR spectrum of the DB gel showed hydrogen‐bonded N–H vibration peak at 3320 cm^−1^ with a C═O stretching vibration signal at 1684 cm^−1^ (Figure S5, red line). To gain insight into the influence of π–π interaction between the central aromatic units upon self‐assembling behaviors, absorption spectra in the solution and aggregated states of **F‐EG_2_‐BU** were compared. The bathochromic effect of the LE absorption band was found in the crystalline film to referenced spectrum of the dilute solution (Figure S6). In the absorption spectrum of the PEG‐600 gel, the peak at around 300 nm slightly red‐shifted against the corresponding peak in the spectrum of the solution state. Because the inclusion of PEG‐600 into the inside of the molecular assembly probably changed the positional relationship between the aromatic cores, the fluctuation in the situation of orbital coupling caused the gaps between each spectrum. Anyhow, the clear difference in the absorption spectra between the dilute solution and PEG‐600 gel implied the presence of core–core interaction. The cooperative effect of π–π interactions and intermolecular hydrogen bonds probably promotes the formation of supramolecular assemblies. Therefore, the SC‐PEG gels probably stabilized these interactions and polar interactions between OEG linkers and SC‐PEGs.

### 2.3 Ion‐Responsive Behaviors

**Figure 6:**
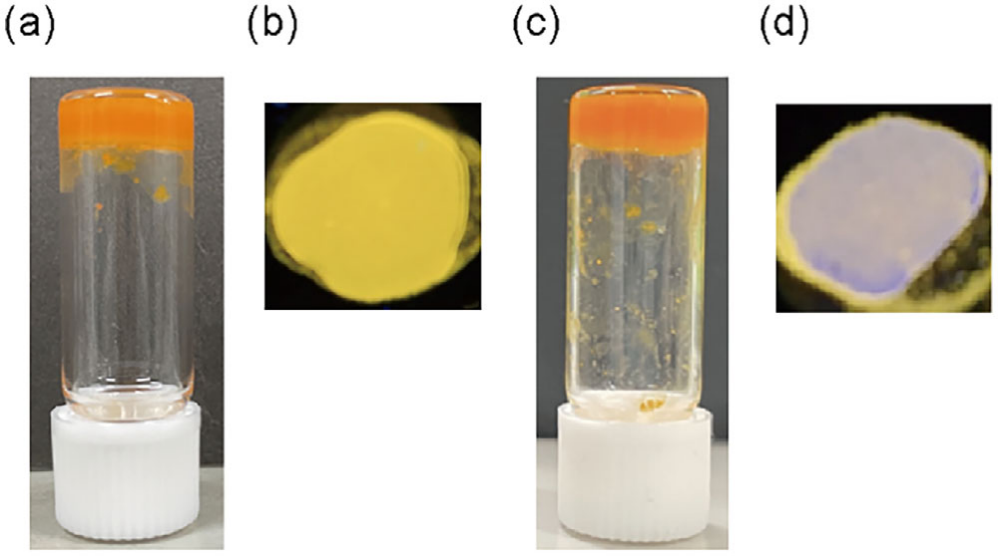
Ion‐responsive behaviors of PEG‐600 gel of **F‐EG_2_‐BU**. Photographs taken (a,b) before and (c,d) after the addition of NaCl (1.0 equiv) under (a,c) the room light and (b,d) UV light (365 nm).

Because complexation can be expected between two diethylene glycol units and one sodium ion,^[^ ^14^ ^]^ the gel of **FU‐EG_2_‐BU** was used to investigate the ion‐responsive behaviors. The ion‐responsive behavior of the PEG‐600 gel of **F‐EG_2_‐BU** is shown in Figure 6. The initial orange‐colored SC‐PEG gel exhibited yellow luminescence (Figure 6b). The gel state of the mixture was maintained upon adding an equivalent molar amount of NaCl in water to the PEG‐600 gel of **F‐EG_2_‐BU** (Figure 6c). NaCl doping of the gel had a minor influence on the light absorption properties (Figure 6a,c; Figure S7a): however, the emission color changed significantly from yellow to purple (Figure 6b,d). The purple photoluminescence of the LE emission was particularly apparent in the emission spectrum of the gel containing NaCl owing to weakened TICT emission (Figure S7b). The water as a solvent for the salt weakened the strength of SC‐PEG gels. However, the mixture sample containing approximately an equivalent molar amount of NaCl with water formed supramolecular gel. The addition of more than 10 equiv molar amount of NaCl caused precipitate. In the SEM image of the xerogel after the addition of an equiv molar amount of NaCl to the PEG‐600 gel of **F‐EG_2_‐BU**, wrinkled sheet‐like assemblies were observed (Figure S12a). The addition of NaCl may have induced wrinkling due to shrinkage. The complexation of sodium cation with OEG linkers and SC‐PEG probably caused the morphological change.

To clarify the influence of halogen ions on the photophysical properties, similar doping experiments were performed using NaBr and NaI as dopants. Although similar reduction in the relative intensity of the TICT emissions from gel samples containing NaBr and NaI were observed (Figure S7b), the largest change in emission intensity was observed in the NaCl‐containing gel. This result indicates the relative contribution of halide anions to the emission properties. The response behaviors of gels containing equivalent amounts of metal chloride salts were also examined to determine the impact of metal cations on the spectroscopic properties. The addition of an equiv amount of KCl or MgCl_2_ to the PEG‐600 gel of **F‐EG_2_‐BU** produces an emission color change (Figure S8b). The variation in the TICT emission in each of these samples was smaller than that in the NaCl‐containing sample: thus, the metal cation also suppressed TICT emission.

**Figure 7:**
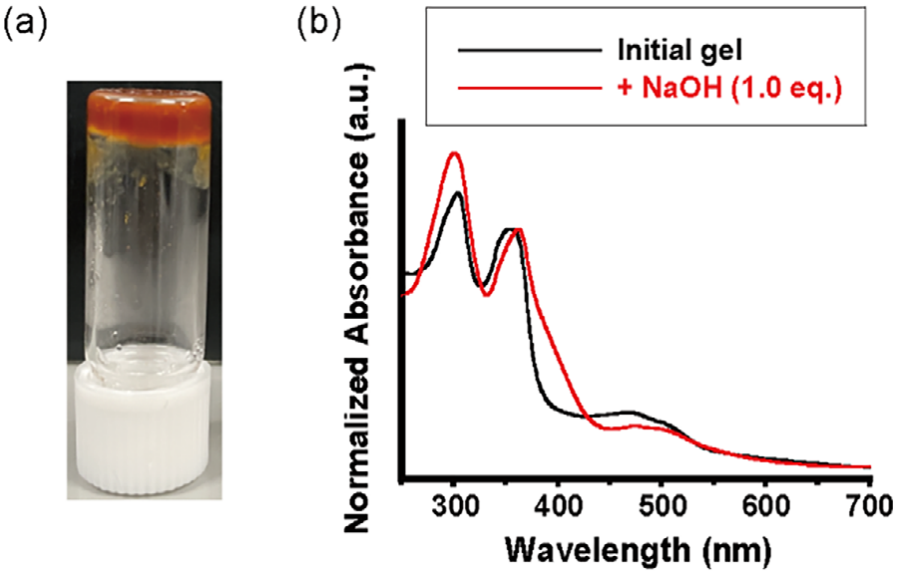
Ion‐responsive behaviors of PEG‐600 gel of **F‐EG_2_‐BU**. (a) Photograph of the gel sample after the addition of NaOH (1.0 equiv) under the room light. (b) Change in absorption spectra of the gel sample after the addition of NaOH (1.0 equiv).

We also examined the variations in the physical properties of the gels in response to other ionic species. A red gel was obtained upon mixing an equivalent amount of NaOH with the PEG‐600 gel of **F‐EG_2_‐BU** (Figure 7a). A shoulder appeared at the base of the LE band in the UV–vis absorption spectrum of the hydroxide‐containing gel, while the second LE and broad ICT bands were redshifted (Figure 7b). The addition of NaOH also influenced the photoluminescence properties of the gel (Figure S9). TICT emission was reduced by the addition of a base. A similar red color was observed upon doping the PEG‐600 gel of **F‐EG_2_‐BU** with KOH rather than NaOH (Figure S9). In the SEM image of the sample after the addition of NaOH, swollen aggregates were observed (Figure S12b).

As the response to NaCl in the PEG‐200 gel of **F‐EG_2_‐BU** was also studied, a similar emission color change was observed (Figure S10). In the PEG‐200 gel system, notably, the relative decrement of TICT emission intensity by the addition of KCl was almost equal to that by the NaCl addition.

To further elucidate the influence of ionic additives on the intermolecular hydrogen bonds in the gel state, the gels containing each ionic species were analyzed using FT‐IR spectroscopy. In the FT‐IR spectra of the gels containing alkali metal halides or hydroxides, peaks assigned to C═O stretching vibrations were observed between approximately 1683–1684 cm^−1^ (Figure S11). Considering these spectroscopic results and the complexation ability of the OEG linker with metal cations,^[^ ^14^, ^15^ ^]^ the stabilization of the planar arrangement and ICT state of π‐conjugated core was attributed to the association of ionic species with the linker, while supramolecular crosslinking was maintained by intermolecular hydrogen bonding.

## 3 Conclusion

Novel D–A–D‐type dye‐based supramolecular gels were developed with ion‐responsive behaviors. A new family of fluorenone‐based D–A–D‐type compounds, incorporating urethane units separated by OEG linkers, was synthesized. These fluorenone derivatives exhibited gelation abilities in SC‐PEGs. **F‐EG_3_‐BU** also gelated ethanol and 2‐propanol. The SC‐PEG gel of **F‐EG_2_‐BU** underwent ion‐responsive absorption and emission color changes, with the most significant emission color change resulting from the addition of NaCl. Furthermore, the color of the SC‐PEG gel changed from orange to red in response to alkali metal hydroxides. Our findings confirm the potential of the D–A–D‐type dye‐based gel system in the colorimetric detection of various chemical stimuli. In this article, we describe a supramolecular gel composed of dye molecules incorporating a D–A–D‐type skeleton and a common solvating medium as a useful and scalable material for constructing stimuli‐responsive materials.

## 4 Experimental

### 4.1 Materials and Synthesis

All reagents were purchased from Tokyo Chemical Industry, Kanto Chemicals, and FUJIFILM Wako Pure Chemical Corporation. All reagents were used as received without further purification. All reactions were performed under an argon atmosphere in a dry three‐necked flask equipped with a magnetic stirring bar.

### 4.2 General Procedures

Unless otherwise stated, the reactions were performed under an argon atmosphere in a dry three‐necked flask equipped with a magnetic stirring bar. All ^1^H and ^13^C NMR spectra were recorded using a Bruker Biospin AVANCE NEO 400 spectrometer at 400 MHz and 100 MHz, respectively. All chemical shifts (δ) in the ^1^H and ^13^C NMR spectra are quoted in ppm using tetramethylsilane (δ = 0.00 ppm) as an internal standard (0.03 vol%). High‐resolution electrospray ionization‐mass spectrometry (HR‐ESI‐MS) was performed using a SCIEX X500R QTOF spectrometer.

### 4.3 Gelation Behaviors and Gel Morphology

The samples were prepared as follows: D–A–D‐type fluorenone derivatives (5 mg) were added to an appropriate volume of organic solvent in 2 mL glass vials. The vials were sealed, and the samples were heated (≈ 110 °C). When a clear solution was obtained upon heating, the mixture was cooled to room temperature (≈ 25 °C). When a vial could be inverted without any flow, the sample was determined to be a “gel”. In the cases where precipitation was observed during cooling or the clear solutions were retained after heating, the samples were denoted “precipitation” and “soluble”, respectively. When the compound could not be dissolved completely even after sufficiently heating, it was determined to be “insoluble. ” The morphologies of the xerogel samples were determined by SEM. The xerogel of **F‐EG_3_‐BU** was prepared from ethanol gel (50 g L^−1^) by freeze–drying. The xerogel sample was coated with gold and platinum by sputtering. SEM was performed in a vacuum using a JEOL JSM‐6510 instrument.

### 4.4 Structural Analysis of Molecular Assemblies

XRD measurements were performed using Ni‐filtered Cu Kα radiation with a Rigaku MiniFlex600 diffractometer. After the crystalline or gel samples were put onto a silicone sample holder, the samples were heated above 110 °C to make their surfaces smooth.

### 4.5 FT‐IR Measurement of Bulk Samples

FT‐IR spectra were obtained using a Thermo Scientific Nicolet iS5 spectrometer in the transmission mode. Bulk samples were prepared on silicone substrates.

### 4.6 Characterization of Spectroscopic Properties in Solution States

The UV–vis absorption spectra of the solution state were recorded using a JASCO V‐650 spectrometer. The UV–vis absorption spectra of dilute solutions (10 µM) were measured using a pair of quartz cells with a cell path length of 1 cm. Photoluminescence emission spectra were recorded in solution using a SHIMADZU RF‐6000 spectrometer. The emission spectra of the dilute solutions (10 µM) were recorded using a quartz cell with a cell path length of 1 cm.

### 4.7 Characterization of Spectroscopic Properties of Gels

Bulk samples were prepared on 1 mm‐thick quartz substrates. The UV–vis absorption spectra of the bulk samples were recorded using a JASCO V‐650 spectrometer. The photoluminescence spectra of the bulk samples were recorded using a Yixi Intelligent Technology YSM‐8101–02–01–16S03L02F06G01 spectrometer. Photoexcitation was achieved using an LED light source (CCS HLV2‐24UV3‐365) with a control unit (CCS PJ2‐1505‐2CA‐PE).

### 4.8 Photoluminescence Quantum Yield

The PLQY was measured using a Hamamatsu Photonics C9920‐02G. The PLQY of the dilute solutions of **F‐EG_2_‐BU**, **F‐EG_3_‐BU,** and **F‐EG_4_‐BU** were measured using a quartz cell with a path length of 1 cm.

### 4.9 Evaluation of Ion‐Responsive Behaviors of Organogels

An appropriate amount (30 µL) of each ionic species in aqueous solution (0.2 M) was added to the SC‐PEG gel of **F‐EG_2_‐BU**. After the mixtures were heated above the gel–sol transition temperature (>110 °C) for homogenization, the samples were left standing and gradually cooled to room temperature. The gelation ability and spectroscopic properties were then examined in the same manner as the bulk samples.

## Supporting Information

Supporting Information is available. The authors have cited additional references in the Supporting Information.^[^ ^16^, ^17^, ^18^ ^]^

## Conflict of Interests

The authors declare no conflict of interest.

## Supporting information



Supporting Information

## Data Availability

The data that support the findings of this study are available in the supplementary material of this article.

## References

[1] a) P. Terech , R. G. Weiss , Chem. Rev. 1997, 97, 3133; 11851487 10.1021/cr9700282

[2] S. Ghosh , V. K. Praveen , A. Ajayaghosh , Annu. Rev. Mater. Res. 2016, 46, 235.

[3] a) D. J. Abdallah , R. G. Weiss , Adv. Mater. 2000, 12, 1237;

[4] a) X. Yan , F. Wang , B. Zheng , F. Huang , Chem. Soc. Rev. 2012, 41, 6042; 22618080 10.1039/c2cs35091b

[5] a) K. Aoki , N. Tamaoki , A. Seki , K. Narazaki , D. Takahashi , K. Horitsugu , Langmuir 2021, 37, 13160; 34706543 10.1021/acs.langmuir.1c02420

[6] a) J.‐L. Pozzo , G. M. Clavier , J.‐P. Desvergne , J. Mater. Chem. 1998, 8, 2575;

[7] N. Sreenivasachary , J.‐M. Lehn , Proc. Natl. Acad. Sci. USA 2005, 102, 5938. 15840720 10.1073/pnas.0501663102 PMC1087938

[8] a) A. Mishra , M. K. R. Fischer , P. Bäuerle , Angew. Chem., Int. Ed. 2009, 48, 2474; 10.1002/anie.200804709 19294671

[9] S. Sasaki , G. P. C. Drummen , G. Konishi , J. Mater. Chem. C 2016, 4, 2731.

[10] a) X. Y. Shen , Y. J. Wang , E. Zhao , W. Z. Yuan , Y. Liu , P. Lu , A. Qin , Y. Ma , J. Z. Sun , B. Z. Tang , J. Phys. Chem. C 2013, 117, 7334;

[11] a) F. Lincker , A.‐J. Attias , F. Mathevet , B. Heinrich , B. Donnio , J.‐L. Fave , P. Rannoua , R. Demadrille , Chem. Commun. 2012, 48, 3209; 10.1039/c2cc17276c 22331231

[12] a) T. D. Thangadurai , C. J. Lee , S. H. Jeong , S. Yoon , Y. G. Seo , Y.‐I. Lee , Microchem. J. 2013, 106, 27;

[13] a) M. Suzuki , A. Seki , S. Yamada , K. Aoki , Mater. Adv. 2024, 5, 7401;

[14] a) S.‐T. Tung , C.‐C. Lai , Y.‐H. Liu , S.‐M. Peng , S.‐H. Chiu , Angew. Chem., Int. Ed. 2013, 52, 13269; 10.1002/anie.201307640 24227458

[15] a) P. G. Bruce , Dalton Trans. 2006, 1365–1369; 16518503 10.1039/b517247k

[16] a) Y. Sagara , Y. C. Simon , N. Tamaoki , C. Weder , Chem. Commun. 2016, 52, 5694; 10.1039/c6cc01614f 27040453

[17] K. E. Murphy‐Benenato , N. Olivier , A. Choy , P. L. Ross , M. D. Miller , J. Thresher , N. Gao , M. R. Hale , ACS Med. Chem. Lett. 2014, 5, 1213. 25408833 10.1021/ml500210x PMC4233352

[18] F. Hu , D. Mao Kenry , X. Cai , W. Wu , D. Kong , B. Liu , Angew. Chem., Int. Ed. 2018, 57, 10182. 10.1002/anie.201805446 29959849

