# Supplementary material for: Color Changes in Gels Composed of Hydrogen‐Bonding Donor–Acceptor–Donor‐Type Fluorenone Derivatives and Short‐Chain Polyethylene Glycol in Response to Ionic Species
Source: Chem Asian J. 2025 Apr 14;20(12):e202500129. doi: 10.1002/asia.202500129 (PMC12204383; doi:10.1002/asia.202500129)
Supplement: Supplementary file 1 — Supporting Information [file ASIA-20-e202500129-s001.pdf]

Supporting Information

Color Changes in Gels Composed of Hydrogen-Bonding Donor–Acceptor–Donor-Type Fluorenone Derivatives and Short-Chain Polyethylene Glycol in Response to Ionic Species

Syota Yamada,[a] Mao Suzuki,[a] Ken'ichi Aoki,*[a, b] and Atsushi Seki*[a, b]

[a] Department of Chemistry, Graduate School of Science, Tokyo University of Science
1-3 Kagurazaka, Shinjuku-ku, Tokyo 162-8601, Japan
E-mail: k-aoki@rs.tus.ac.jp (K.A.), a_seki_3@rs.tus.ac.jp (A.S.)

[b] Department of Chemistry, Faculty of Science, Tokyo University of Science
1-3 Kagurazaka, Shinjuku-ku, Tokyo 162-8601, Japan

1. **Synthesis**
2. **Light Absorption and Emission Properties**
3. **Morphology of Gels**
4. **Structural Analysis of Molecular Assemblies**
5. **FT-IR Spectra of Molecular Assemblies**
6. **Light Absorption Properties in Gel State**
7. **Ion-Response Behaviors**
8. **^{1}H and ^{13}C NMR Spectra**
9. **HR-ESI-MS Spectra**

1. Synthesis

The D–A–D-type fluorenone-based bisurethanes **F-EG$_2$-BU**, **F-EG$_3$-BU** and **F-EG$_4$-BU** were synthesized as shown in Scheme S1. The tetrahydropyranyl-protected D–A–D compound **1** was synthesized via the Sonogashira-Hagihara reaction between 2,7-dibromo-9-fluorenone and 2-(4-ethynylphenoxy)tetrahydro-2*H*-pyran.[16] The deprotection by hydrochloric acid gave the precursor **2**.[17] The Williamson ether synthesis reactions between **2** and oligo(ethylene glycol) monotosylate[18] were afforded hydroxy-terminated compounds **3-EG$_2$**, **3-EG$_3$** and **3-EG$_4$**, respectively. Each target compounds **F-EG$_2$-BU**, **F-EG$_3$-BU** and **F-EG$_4$-BU** were synthesized via urethanation between hexylisocyanate and corresponding hydroxy compounds (**3-EG$_2$**, **3-EG$_3$** or **3-EG$_4$**).

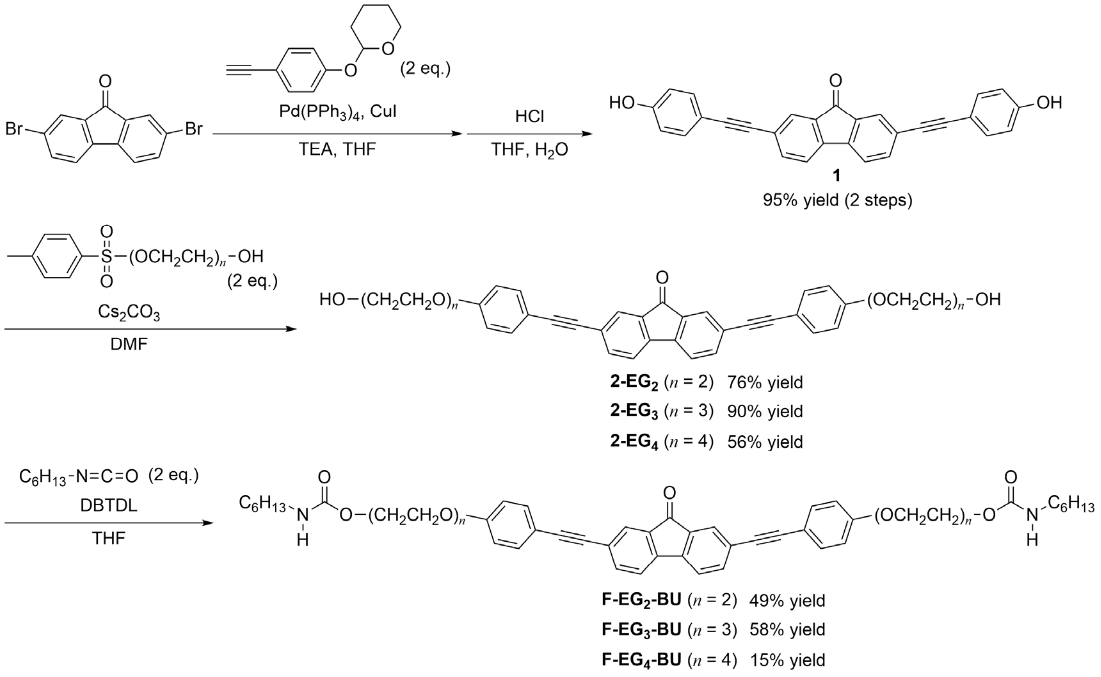

Scheme S1. Synthetic route of hydrogen-bonding D–A–D-type fluorenone derivatives.

Compound **1**

To a stirred suspension of 2,7-dibromo-9-fluorenone (1.04 g, 3.07 mmol, 1.00 eq.), 2-(4-ethynylphenoxy)tetrahydro-2*H*-pyran (1.58 g, 7.58 mmol, 2.46 eq.), CuI (36.8 mg, 0.20 mmol, 0.07 eq.), and Pd(PPh$_3$)$_4$ (181 mg, 0.16 mmol, 0.05 eq.) in dry tetrahydrofuran (THF, 40 mL) and was added triethylamine (TEA, 30 mL). The mixture was refluxed for 24 hours. After monitoring the reaction progress by TLC, the mixture was cooled to room temperature. The reaction mixture was filtered to remove the insoluble residue. The volatile components were evaporated under reduced pressure from the filtrate. Then, the resultant residue was pathed through a short silica gel column (eluent: THF/chloroform = 10/1; v/v). The obtained crude product was used for the next reaction without further purification. To a stirred mixture of crude THP-protected compound and THF (40 mL), 10wt% HCl (3

mL) was added. Then, the mixture was stirred at room temperature for over 5 hours. After monitoring the reaction progress by TLC, the reaction mixture was diluted by water (50 mL). The product was extracted with chloroform (40 mL ×2). The collected organic fractions were combined and dried over anhydrous Na_2SO_4. After filtration and evaporation, the resultant residue was purified by silica gel column chromatography (eluent: toluene/THF = 10/1; v/v). The product was recrystallized from THF/*n*-hexane. The separated solid was dried *in vacuo* to afford deprotected compound **1** as a red solid (1.21 g, 2.92 mmol, 95% yield in 2 steps).

^{1}H NMR (400 MHz, acetone-D_6): δ [ppm] = 7.88–7.82 (m, 2H), 7.78–7.71 (m, 4H), 7.45 (d, *J* = 8.8 Hz, 4H), 6.90 (d, *J* = 8.8 Hz, 4H).

2-EG$_2$

The mixture of compound **1** (471 mg, 1.14 mmol, 1.00 eq.), diethylene glycol mono-(*p*-toluenesulfonate) (970 mg, 3.73 mmol, 3.27 eq.) and cesium carbonate (1.53 g, 4.69 mmol, 4.11 eq.) in *N*,*N*-dimethylformamide (DMF, 50 mL) was stirred at 80 C for 29 hours. The crude product was extracted with chloroform (100 mL) and washed with sat. NH_4Cl aq. (50 mL ×5). The collected organic fractions were combined and dried over anhydrous Na_2SO_4. After filtration and evaporation, the resultant residue was purified by recrystallization from THF/*n*-hexane. The separated cake was dried *in vacuo* to afford **2-EG$_2$** as an orange crystal (510 mg, 0.87 mmol, 76% yield).

^{1}H NMR (400 MHz, $CDCl_3$): δ [ppm] = 7.79–7.76 (m, 2H), 7.64–7.60 (m, 2H), 7.52–7.42 (m, 6H), 6.91 (d, *J* = 8.8 Hz, 4H), 4.17 (t, *J* = 4.4 Hz, 4H), 3.88 (t, *J* = 4.8 Hz, 4H), 3.77–3.69 (m, 12H), 3.65–3.61 (m, 4H).

2-EG$_3$

The mixture of compound **1** (400 mg, 0.97 mmol, 1.00 eq.), diethylene glycol mono-(*p*-toluenesulfonate) (950 mg, 3.12 mmol, 3.21 eq.) and cesium carbonate (1.21 g, 3.70 mmol, 3.81 eq.) in DMF (50 mL) was stirred at 80 C for 24 hours. The crude product was extracted with chloroform (100 mL) and washed with sat. NH_4Cl aq. (50 mL ×3). The collected organic fractions were combined and dried over anhydrous Na_2SO_4. After filtration and evaporation, the resultant residue was purified by recrystallization from THF/*n*-hexane. The separated crystal was dried *in vacuo* to afford **2-EG$_3$** as an orange solid (592 mg, 0.87 mmol, 90% yield).

^{1}H NMR (400 MHz, $CDCl_3$): δ [ppm] = 7.79–7.76 (m, 2H), 7.64–7.60 (m, 2H), 7.52–7.42 (m, 6H), 6.91 (d, *J* = 8.8 Hz, 4H), 4.17 (t, *J* = 4.4 Hz, 4H), 3.88 (t, *J* = 4.8 Hz, 4H), 3.77–3.69 (m, 12H), 3.65–3.61 (m, 4H).

2-EG$_4$

The mixture of compound **1** (407 mg, 0.99 mmol, 1.00 eq.), tetraethylene glycol mono-(*p*-toluenesulfonate) (1.24 g, 3.56 mmol, 3.60 eq.) and cesium carbonate (1.25 g, 3.84 mmol, 3.88 eq.) in DMF (40 mL) was stirred at 85 C for 20 hours. The crude product was extracted with chloroform (100

mL) and washed with sat. NH_4Cl aq. (50 mL ×3). The collected organic fractions were combined and dried over anhydrous Na_2SO_4. After filtration and evaporation, the resultant residue was purified by recrystallization from THF/*n*-hexane. The separated crystal was dried *in vacuo* to afford **2-EG$_4$** as a yellow solid (420 mg, 0.55 mmol, 56% yield).

^{1}H NMR (400 MHz, $CDCl_3$): δ [ppm] = 7.80–7.58 (m, 2H), 7.66–7.58 (m, 2H), 7.52–7.42 (m, 6H), 6.90 (d, *J* = 8.8 Hz, 4H), 4.25–4.12 (m, 4H), 3.93–3.82 (m, 4H), 3.79–3.63 (m, 22H), 3.63–3.59 (m, 4H).

F-EG$_2$-BU

To a stirred solution of **3-EG$_2$** (397 mg, 0.67 mmol, 1.00 eq.) in dry THF (35 mL) was added hexyl isocyanate (500 μL, 3.46 mmol, 5.16 eq.) at room temperature. Then, dibutyltin dilaurate (120 μL, 0.28 mmol, 0.42 eq.) was immediately added to the reaction mixture. The reaction mixture was stirred overnight at room temperature. After monitoring the reaction progress by TLC, the crude product was extracted with chloroform (100 mL) and washed with H_2O (100 mL ×2). The collected organic fractions were combined and dried over anhydrous Na_2SO_4. After filtration and evaporation, the resultant residue was recrystallized from THF/methanol several times. The obtained solid was dried *in vacuo* to afford **F-EG$_2$-BU** as an orange crystal (279 mg, 0.33 mmol, 49% yield).

^{1}H NMR (400 MHz, $CDCl_3$): δ [ppm] = 7.76 (d, *J* = 1.0 Hz, 2H), 7.61 (dd, *J* = 7.7, 1.5 Hz, 2H), 7.50–7.43 (m, 6H), 6.90 (d, *J* = 8.8 Hz, 4H), 4.73 (br-s, 2H), 4.25 (t, *J* = 4.4 Hz, 4H), 4.15 (t, *J* = 4.8 Hz, 4H), 3.86 (t, *J* = 4.8 Hz, 4H), 3.76 (t, *J* = 4.8 Hz, 4H), 3.15 (quartet, *J* = 6.4 Hz, 4H), 1.50–1.42 (m, 4H), 1.36–1.23 (m, 12H), 0.88 (t, *J* = 4.0 Hz, 6H); ^{13}C NMR (100 MHz, $CDCl_3$): δ [ppm] = 192.4, 159.1, 156.3, 143.0, 137.6, 134.4, 133.2, 127.2, 124.8, 120.5, 115.1, 114.7, 91.5, 87.5, 70.0, 69.5, 67.5, 63.7, 41.1, 31.5, 29.9, 26.4, 22.6, 14.0; HRMS (ESI): molecular weight: 843.0300 ($C_{51}H_{58}N_2O_9$); *m*/*z* calcd. for $[C_{51}H_{59}N_2O_9]^+$ ($[M+H]^+$): 843.4215; *m*/*z* found. 843.4209.

F-EG$_3$-BU

To a stirred solution of **3-EG$_3$** (404 mg, 0.60 mmol, 1.00 eq.) in dry THF (45 mL) was added hexyl isocyanate (500 μL, 3.46 mmol, 5.76 eq.) at room temperature. Then, dibutyltin dilaurate (100 μL, 0.23 mmol, 0.39 eq.) was immediately added to the reaction mixture. The reaction mixture was stirred overnight at room temperature. After monitoring the reaction progress by TLC, the crude product was diluted with chloroform (130 mL). The solution was washed with sat. NaCl aq. (100 mL ×1) and H_2O (100 mL ×3). The collected organic fractions were combined and dried over anhydrous Na_2SO_4. After filtration and evaporation, the resultant residue was purified by recrystallization from THF/*n*-hexane. The obtained solid was further purified by recrystallization from THF/methanol two times. The collected solid was dried *in vacuo* to afford **F-EG$_3$-BU** as an orange solid (317 mg, 0.35 mmol, 58% yield).

^{1}H NMR (400 MHz, $CDCl_3$): δ [ppm] = 7.77 (dd, *J* = 1.5, 0.5 Hz, 2H), 7.62 (dd, *J* = 7.7, 1.5 Hz, 2H), 7.51–7.43 (m, 6H), 6.90 (d, *J* = 9.2 Hz, 4H), 4.74 (br-s, 2H), 4.22 (t, *J* = 4.4 Hz, 4H), 4.15 (t, *J* = 4.4 Hz, 4H), 3.87 (t, *J* = 4.4 Hz, 4H), 3.76–3.66 (m, 12H), 3.15 (quartet, *J* = 6.4 Hz, 4H), 1.52–1.41 (m, 4H),

1.37–1.21 (m, 12H), 0.88 (t, *J* = 6.8 Hz, 6H); ^{13}C NMR (100 MHz, $CDCl_3$): δ [ppm] = 192.5, 159.1, 156.4, 143.0, 137.6, 134.4, 133.2, 127.2, 124.8, 120.5, 115.1, 114.8, 91.5, 87.5, 70.9, 70.6, 69.8, 69.7, 67.5, 63.8, 41.1, 31.5, 29.9, 26.4, 22.6, 14.0; HRMS (ESI): molecular weight: 931.1360 ($C_{55}H_{66}N_2O_{11}$); *m*/*z* calcd. for $[C_{55}H_{67}N_2O_{11}]^+$ ($[M+H]^+$): 931.4739; *m*/*z* found. 931.4726.

F-EG$_4$-BU

To a stirred solution of **3-EG$_4$** (260 mg, 0.34 mmol, 1.00 eq.) in dry THF (30 mL) was added hexyl isocyanate (250 μL, 1.70 mmol, 5.00 eq.) at room temperature. Then, dibutyltin dilaurate (60 μL, 0.14 mmol, 0.40 eq.) was immediately added to the reaction mixture. The reaction mixture was stirred overnight at room temperature. After monitoring the reaction progress by TLC, the crude product was extracted with chloroform (100 mL) and washed with water (100 mL ×3). The collected organic fractions were combined and dried over anhydrous Na_2SO_4. After filtration and evaporation, the resultant residue was purified by silica gel column chromatography (eluent: chloroform/THF = 6/1; v/v). The product was recrystallized from THF/methanol two times. The obtained solid was dried *in vacuo* to afford **F-EG$_4$-BU** as an orange solid (52.2 mg, 0.05 mmol, 15% yield).

^{1}H NMR (400 MHz, $CDCl_3$): δ [ppm] = 7.78 (dd, *J* = 1.5, 0.6 Hz, 2H), 7.63 (dd, *J* = 7.7, 1.5 Hz, 2H), 7.49 (dd, *J* = 7.7, 0.6 Hz, 2H), 7.46 (d, *J* = 8.8 Hz, 4H), 6.91 (d, *J* = 8.9 Hz, 4H), 4.76 (br-s, 2H), 4.22 (t, *J* = 4.6 Hz, 4H), 4.16 (t, *J* = 4.9 Hz, 4H), 3.87 (t, *J* = 4.8 Hz, 4H), 3.76–3.63 (m, 20H), 3.15 (quartet, *J* = 6.7 Hz, 4H), 1.55–1.40 (m, 4H), 1.35–1.22 (m, 12H), 0.88 (t, *J* = 6.9 Hz, 6H); ^{13}C NMR (100 MHz, $CDCl_3$): δ [ppm] = 192.5, 159.2, 156.4, 143.0, 137.6, 134.5, 133.2, 127.3, 124.8, 120.5, 115.0, 114.8, 91.5, 87.5, 70.9, 70.7, 70.6, 70.5, 69.7, 69.7, 67.5, 63.8, 41.1, 31.5, 29.9, 26.4, 22.6, 14.0; HRMS (ESI): molecular weight: 1019.2420 ($C_{59}H_{74}N_2O_{13}$); *m*/*z* calcd. for $[C_{59}H_{75}N_2O_{13}]^+$ ($[M+H]^+$): 1019.5264; *m*/*z* found. 1019.5245.

2. Light Absorption and Emission Properties

Table S1. Photophysical properties of hydrogen-bonding D–A–D-type fluorenone derivatives in THF (10 μM).

Compound	λ_{abs} / nm	λ_{em} / nm[a]	PLQY[b]
**F-EG$_2$-BU**	302, 358, 451	405, 552	0.297
**F-EG$_3$-BU**	302, 358, 450	408, 551	0.217
**F-EG$_4$-BU**	302, 358, 448	407, 561	0.208

[a] The excitation wavelength was set at the absorption maximum around 350 nm.

[b] The excitation wavelength is 365 nm.

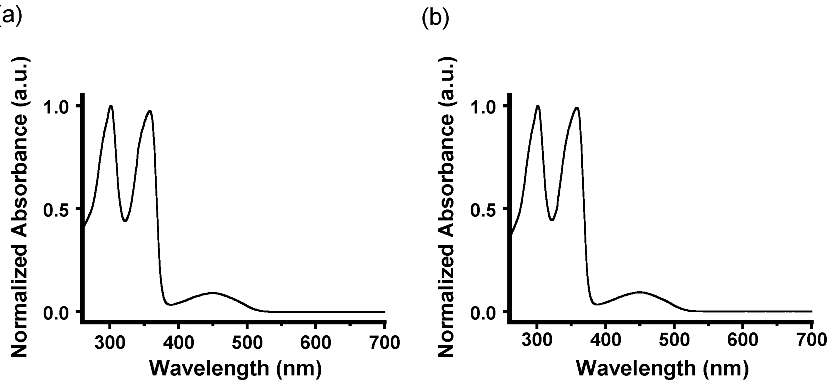

Figure S1. Absorption spectra of (a) **F-EG$_3$-BU** and (b) **F-EG$_4$-BU** in THF (10 μM).

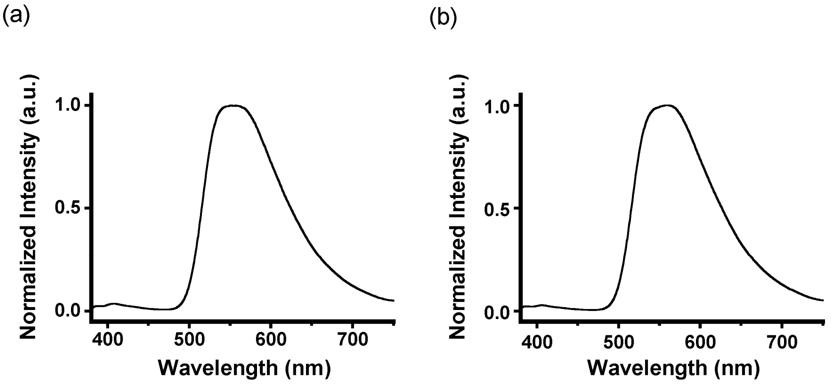

Figure S2. Emission spectra of (a) **F-EG$_3$-BU** and (b) **F-EG$_4$-BU** in THF (10 μM).

3. Morphology of Gels

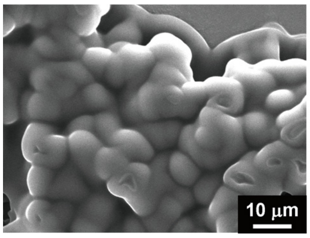

Figure S3. An SEM image of the swollen aggregates in PEG-600 gel of **F-EG$_3$-BU**.

4. Structural Analysis of Molecular Assemblies

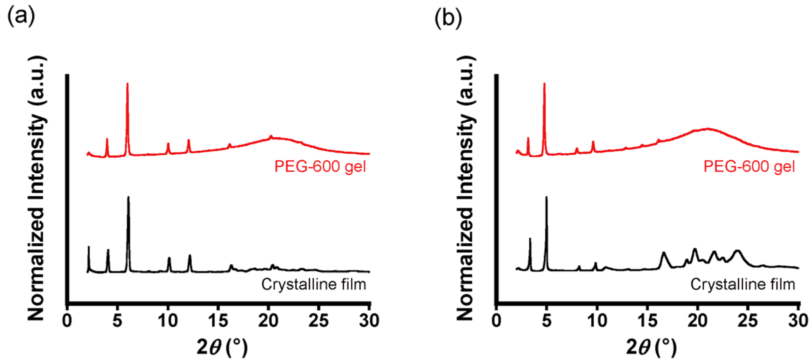

Figure S4. XRD patterns of PEG-600 gel and crystalline film for (a) **F-EG$_2$-BU** and (b) **F-EG$_3$-BU**.

5. FT-IR Spectra of Molecular Assemblies

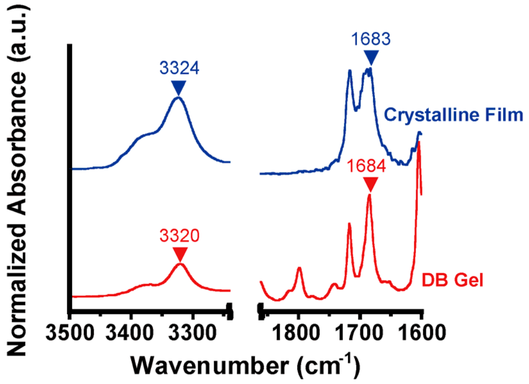

Figure S5. FT-IR spectra of **F-EG$_2$-BU** in DB gel and crystalline film.

6. Light Absorption Properties in Gel State

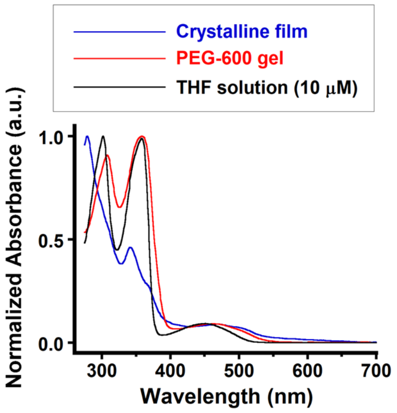

Figure S6. The absorption spectra in the THF solution (10 μM), PEG-600 gel and crystalline film states of **F-EG$_2$-BU**.

7. Ion-Response Behaviors

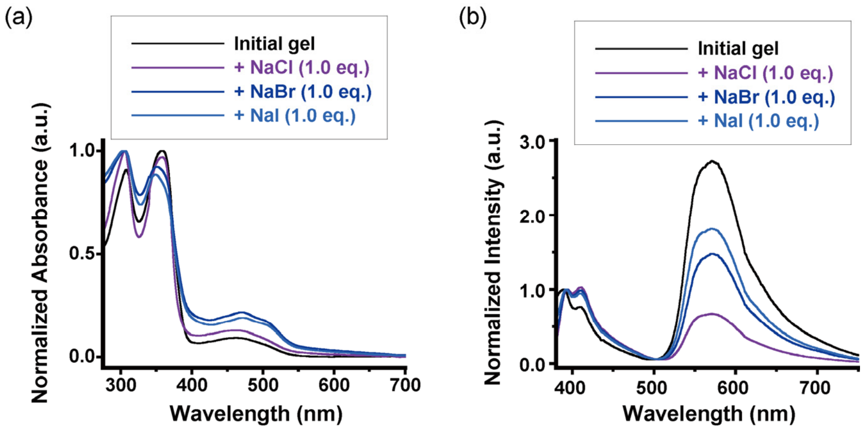

Figure S7. The variation of (a) absorption and (b) emission spectra in the PEG-600 gels for **F-EG$_2$-BU** by the addition of sodium halide salts.

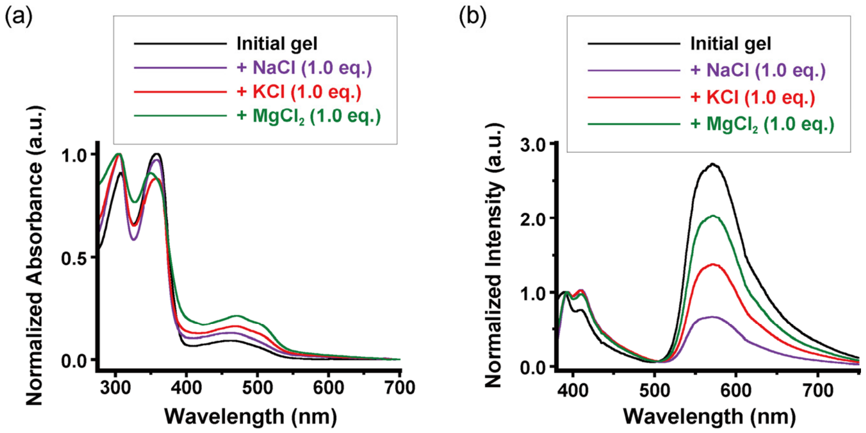

Figure S8. The variation of (a) absorption and (b) emission spectra in the PEG-600 gels for **F-EG$_2$-BU** by the addition of metal chloride salts.

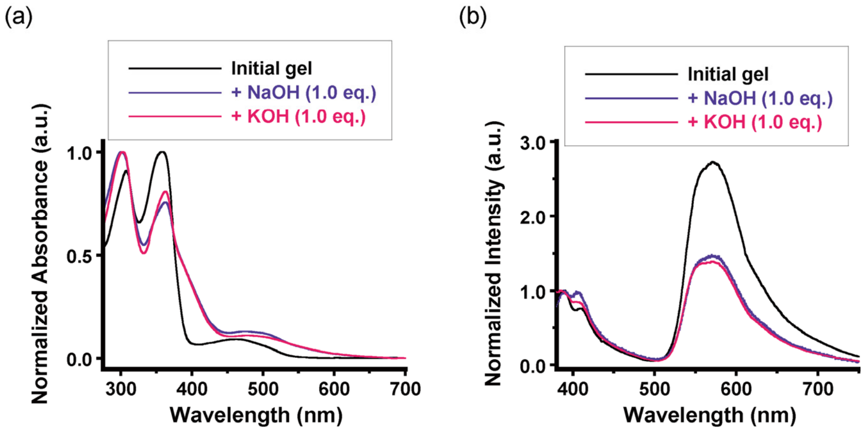

Figure S9. The variation of (a) absorption and (b) emission spectra in the PEG-600 gels for **F-EG$_2$-BU** by the addition of metal hydroxides.

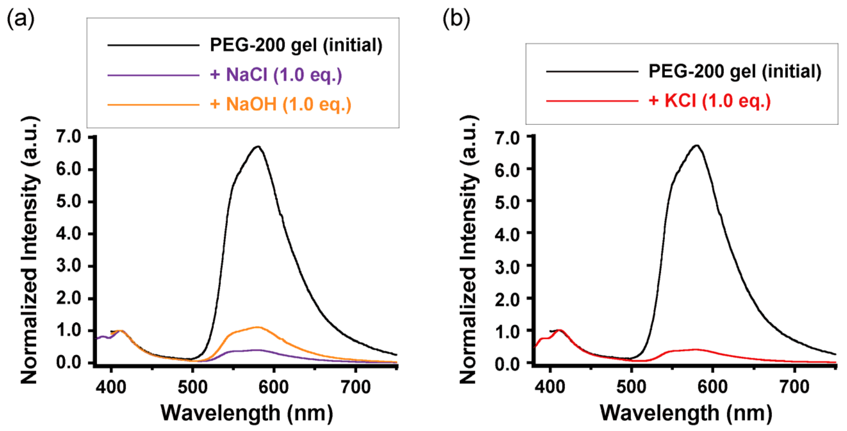

Figure S10. The variation of emission spectra in the PEG-200 gels for **F-EG$_2$-BU** by the addition of (a) sodium chloride and sodium hydroxide, and (b) potassium chloride.

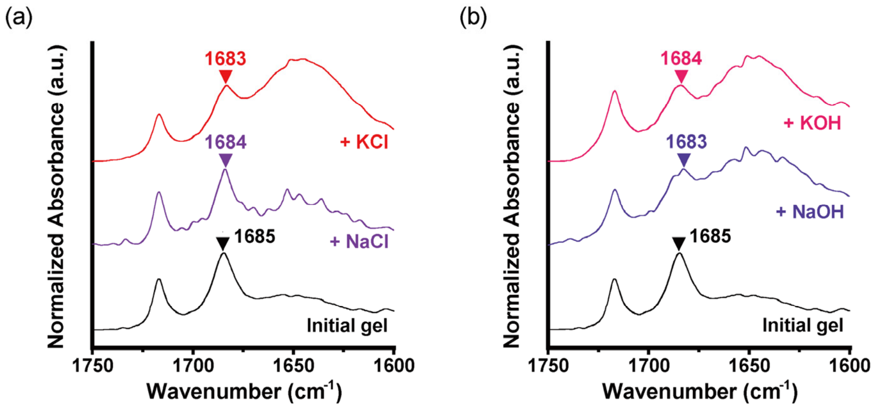

Figure S11. The variation of FT-IR spectra in the PEG-600 gels for **F-EG$_2$-BU** by the addition of (a) alkali-metal halides and (b) alkali-metal hydroxides.

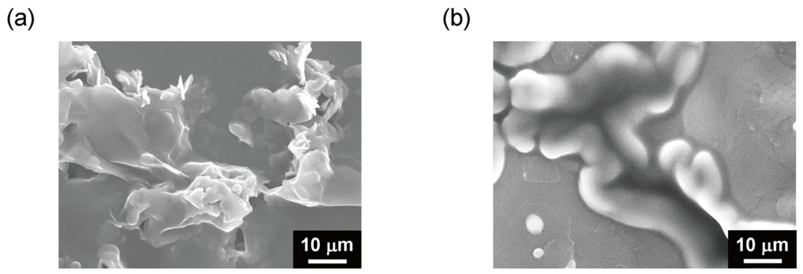

Figure S12. SEM images of (a) the xerogel of **F-EG$_2$-BU** prepared from SC-PEG gel containing an equivalent amount of NaCl, and (b) the swollen aggregates in SC-PEG gel of **F-EG$_2$-BU** after the addition of an equivalent amount of NaOH.

8. ¹H and ¹³C NMR Spectra

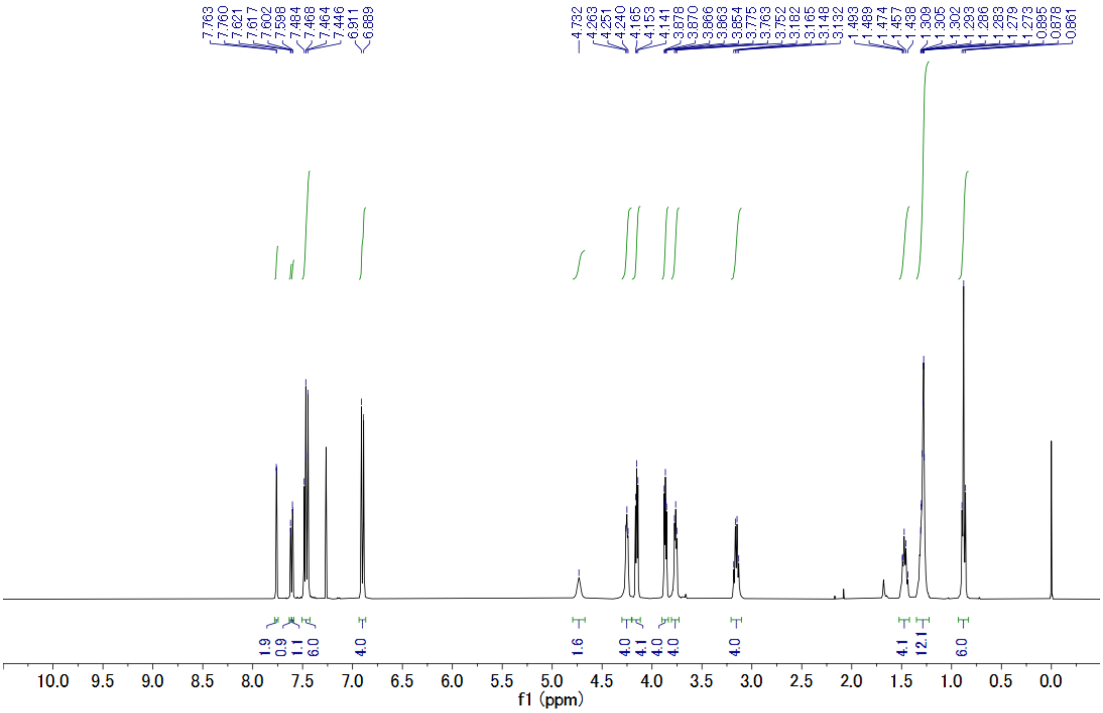

Figure S13. ¹H NMR spectrum of **F-EG₂-BU**.

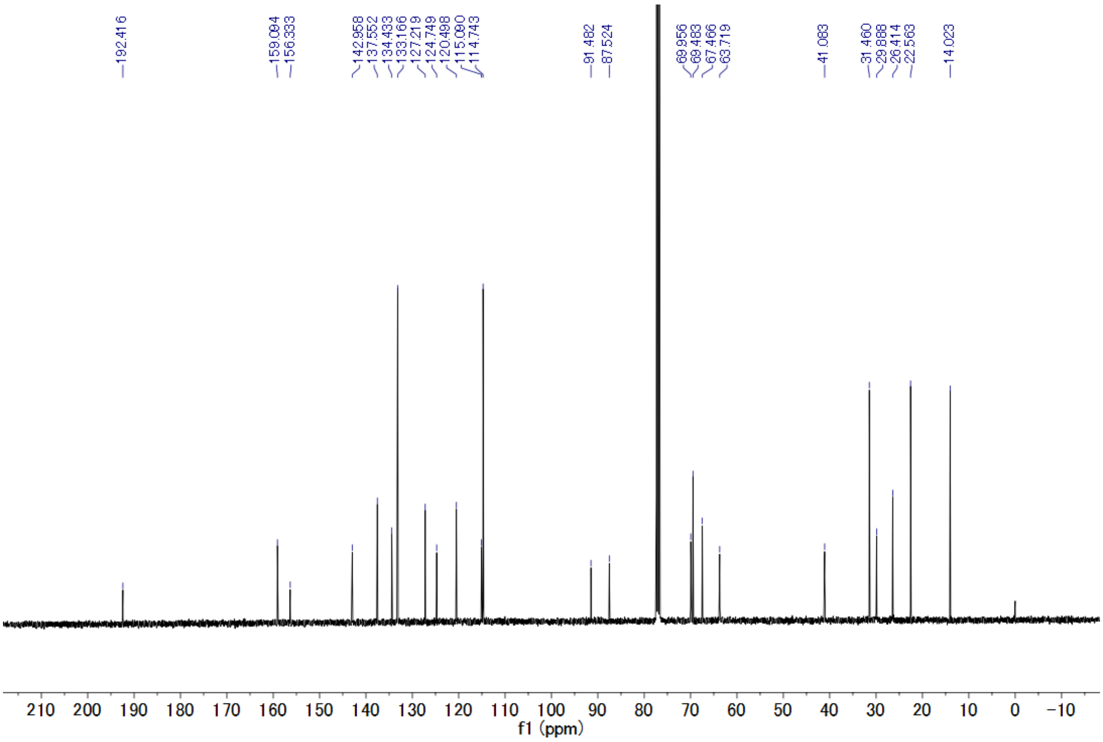

Figure S14. ¹³C NMR spectrum of **F-EG₂-BU**.

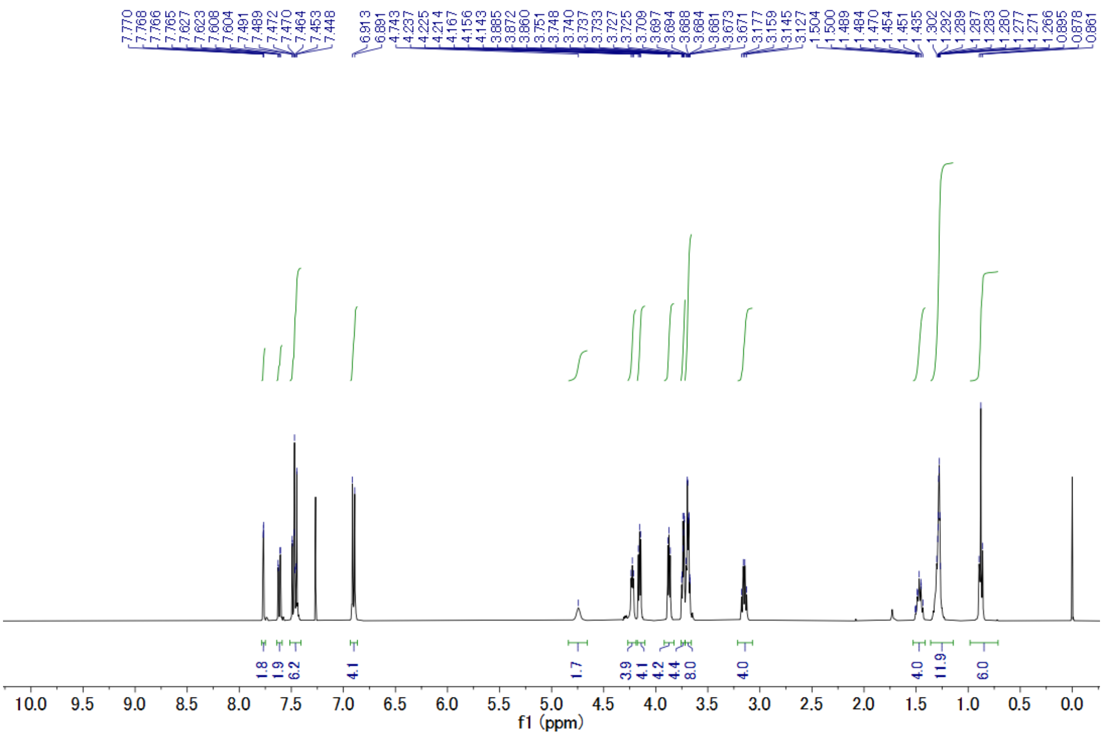

Figure S15. ^{1}H NMR spectrum of **F-EG$_3$-BU**.

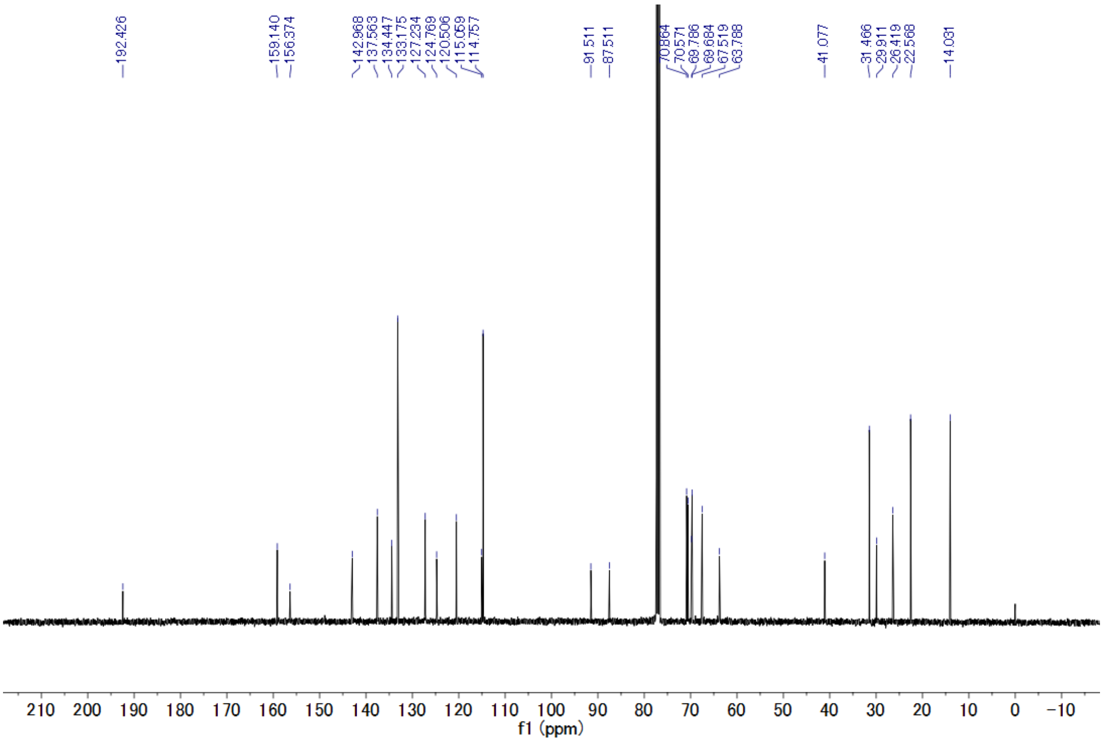

Figure S16. ^{13}C NMR spectrum of **F-EG$_3$-BU**.

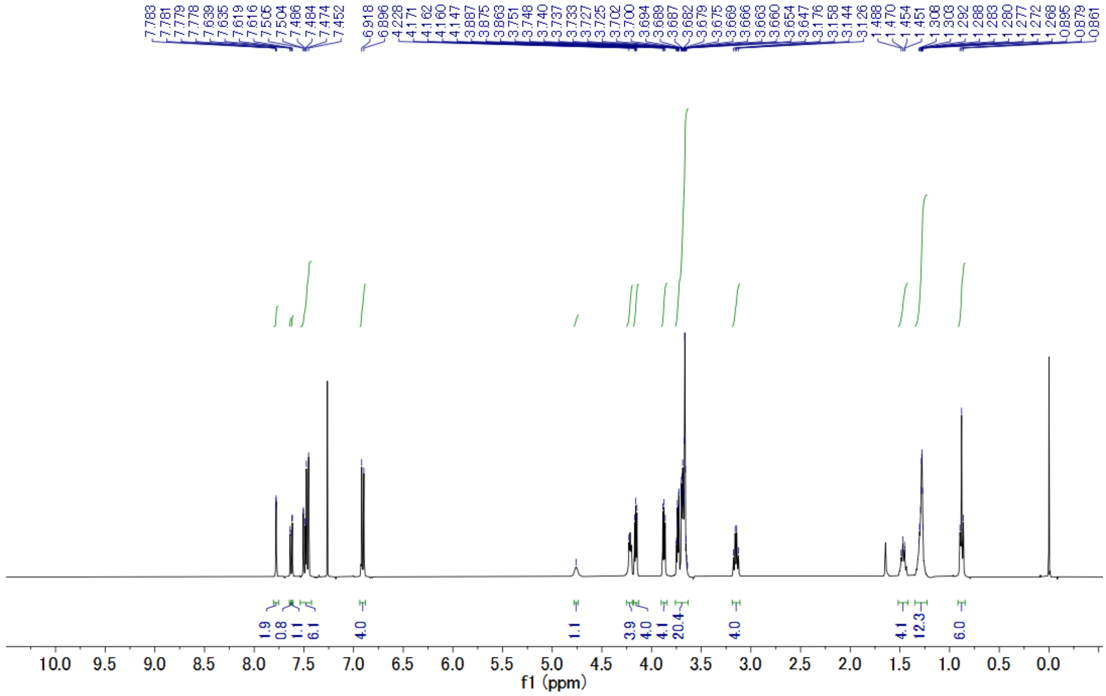

Figure S17. ^{1}H NMR spectrum of **F-EG$_4$-BU**.

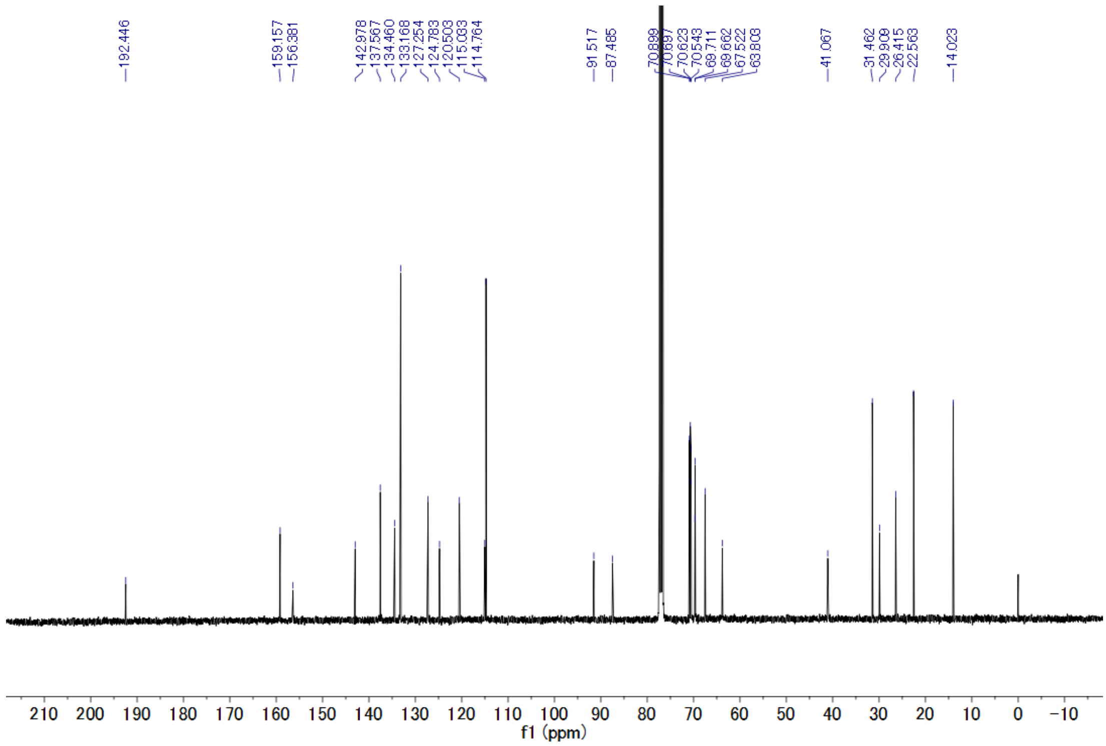

Figure S18. ^{13}C NMR spectrum of **F-EG$_4$-BU**.

9. HR-ESI-MS Spectra

Formula $[M+H]^+$	Theoretical *m/z*	Found *m/z*	RDB	Delta *m/z* (ppm)	Resolution
$C_{51}H_{59}N_2O_9$	843.4215	843.4209	24.0	-0.7	30590

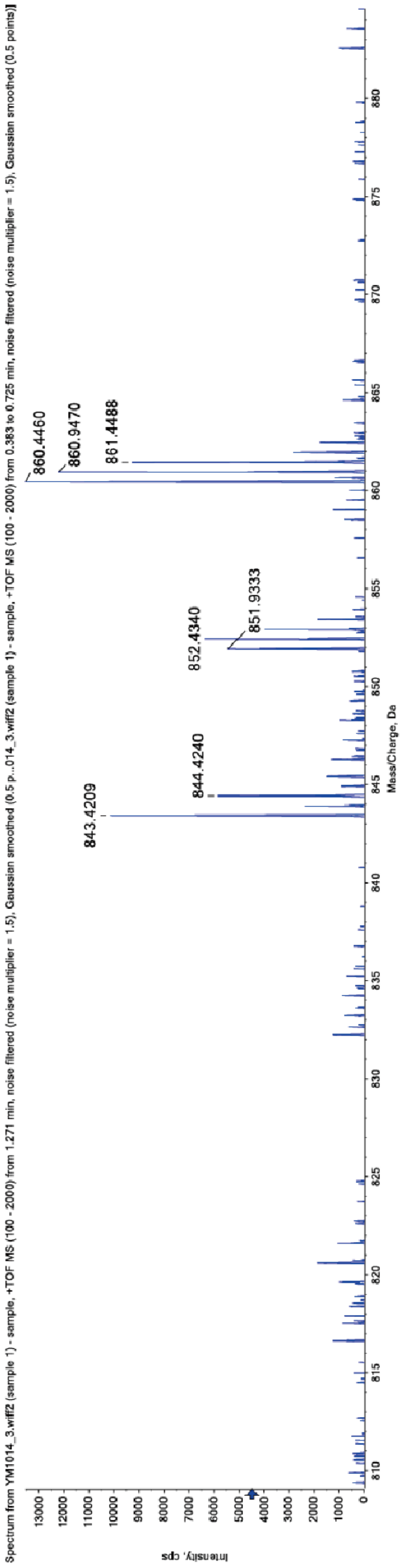

Figure S19. HR-ESI-MS spectrum of **F-EG$_2$-BU**.

Formula $[M+H]^+$	Theoretical *m/z*	Found *m/z*	RDB	Delta *m/z* (ppm)	Resolution
$C_{55}H_{67}N_2O_{11}$	931.4739	931.4726	24.0	-1.4	32567

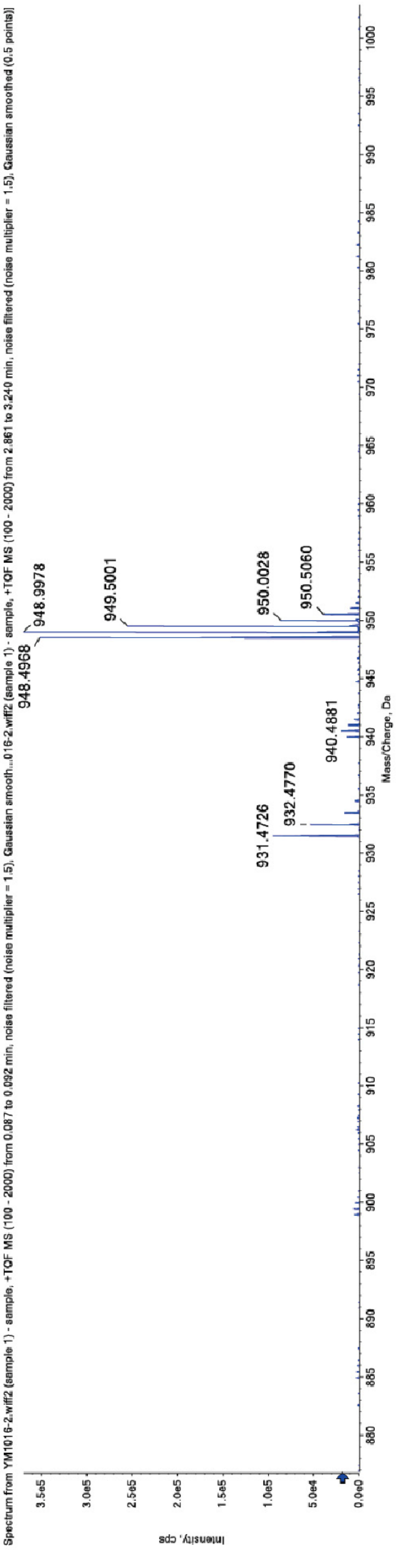

Figure S20. HR-ESI-MS spectrum of **F-EG$_3$-BU**.

Formula $[M+H]^+$	Theoretical *m/z*	Found *m/z*	RDB	Delta *m/z* (ppm)	Resolution
$C_{59}H_{75}N_2O_{13}$	1019.5264	1019.5245	24.0	-1.8	37507

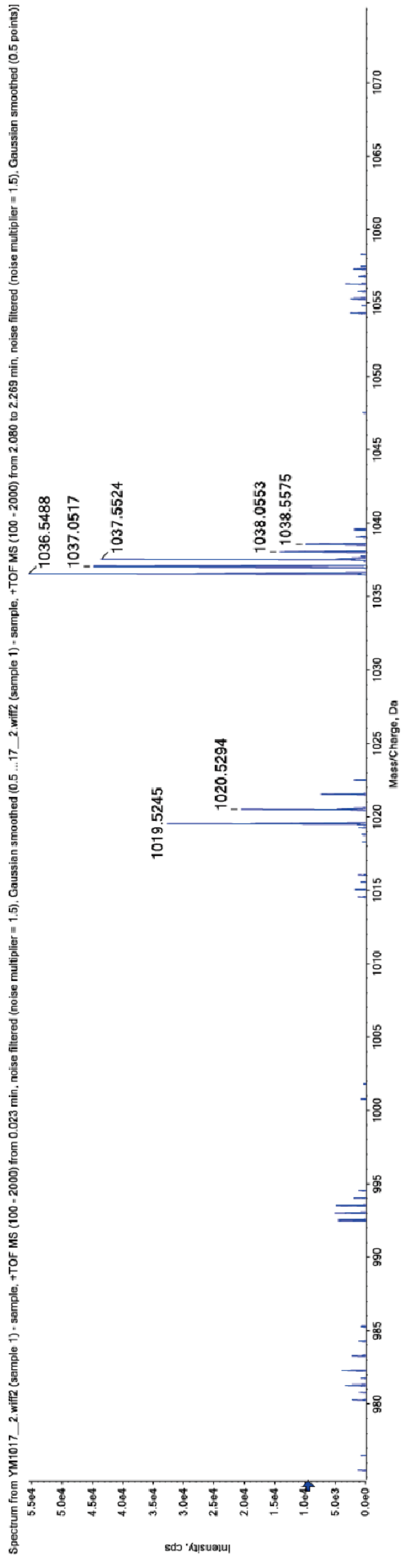

Figure S21. HR-ESI-MS spectrum of **F-EG$_4$-BU**.
